# CsGDH2.1 negatively regulates theanine accumulation in late-spring tea plants (*Camellia sinensis* var. *sinensis*)

**DOI:** 10.1093/hr/uhac245

**Published:** 2022-11-03

**Authors:** Tingting Chen, Jingzhen Ma, Huiping Li, Shijia Lin, Chunxia Dong, Yunxia Xie, Xiaomei Yan, Shupei Zhang, Tianyuan Yang, Xiaochun Wan, Zhaoliang Zhang

**Affiliations:** State Key Laboratory of Tea Plant Biology and Utilization, Anhui Agricultural University, Hefei 230036, China; State Key Laboratory of Tea Plant Biology and Utilization, Anhui Agricultural University, Hefei 230036, China; State Key Laboratory of Tea Plant Biology and Utilization, Anhui Agricultural University, Hefei 230036, China; State Key Laboratory of Tea Plant Biology and Utilization, Anhui Agricultural University, Hefei 230036, China; State Key Laboratory of Tea Plant Biology and Utilization, Anhui Agricultural University, Hefei 230036, China; State Key Laboratory of Tea Plant Biology and Utilization, Anhui Agricultural University, Hefei 230036, China; State Key Laboratory of Tea Plant Biology and Utilization, Anhui Agricultural University, Hefei 230036, China; State Key Laboratory of Tea Plant Biology and Utilization, Anhui Agricultural University, Hefei 230036, China; State Key Laboratory of Tea Plant Biology and Utilization, Anhui Agricultural University, Hefei 230036, China; State Key Laboratory of Tea Plant Biology and Utilization, Anhui Agricultural University, Hefei 230036, China; State Key Laboratory of Tea Plant Biology and Utilization, Anhui Agricultural University, Hefei 230036, China

## Abstract

Theanine, a unique and the most abundant non-proteinogenic amino acid in tea plants, endows tea infusion with the umami taste and anti-stress effects. Its content in tea correlates highly with green tea quality. Theanine content in new shoots of tea plants is high in mid-spring and greatly decreases in late spring. However, how the decrease is regulated is largely unknown. In a genetic screening, we observed that a yeast mutant, *glutamate dehydrolase 2* (*gdh2*), was hypersensitive to 40 mM theanine and accumulated more theanine. This result implied a role of CsGDH2s in theanine accumulation in tea plants. Therefore, we identified the two homologs of GDH2, CsGDH2.1 and CsGDH2.2, in tea plants. Yeast complementation assay showed that the expression of *CsGDH2.1* in yeast *gdh2* mutant rescued the theanine hypersensitivity and hyperaccumulation of this mutant. Subcellular localization and tissue-specific expression showed CsGDH2.1 localized in the mitochondria and highly expressed in young tissues. Importantly, *CsGDH2.1* expression was low in early spring, and increased significantly in late spring, in the new shoots of tea plants. These results all support the idea that CsGDH2.1 regulates theanine accumulation in the new shoots. Moreover, the *in vitro* enzyme assay showed that CsGDH2.1 had glutamate catabolic activity, and knockdown of *CsGDH2.1* expression increased glutamate and theanine accumulation in the new shoots of tea plants. These findings suggested that CsGDH2.1-mediated glutamate catabolism negatively regulates theanine accumulation in the new shoots in late spring, and provides a functional gene for improving late-spring green tea quality.

## Introduction

The tea plant (*Camellia sinensis*) is one of the most important cash crops in the world, and is widely cultivated in more than 60 countries. The new shoots of tea plants are plucked to be processed into teas. Tea contains abundant secondary metabolites, such as catechins, caffeine, and theanine. These metabolites determine both the sensory quality and the health benefits of tea [1]. Among these tea quality-conferring components, theanine is the most characteristic one.

It is a tea plant-specific non-proteinogenic amino acid and highly accumulates in tea plants, accounting for 1%–2% of dry weight and >50% of total free amino acids in the new shoots [2, 3]. Theanine endows tea infusion with the umami taste and counteracts the bitterness and astringency of caffeine, catechins and catechin derivatives in green tea [4]. Apart from its role in green tea flavor, theanine has also been reported to have benefits for human health, such as having an anti-stress effect, improving learning ability and memory, preventing cardiovascular disease, and protecting against cancer [5, 6]. Therefore, the theanine content is highly correlated with green tea quality [7].

Theanine biosynthesis from glutamate and ethylamine mainly occurs in the roots of tea plants and is catalyzed by theanine synthetase CsTSI [ 2, 8–10]. Glutamine synthetases (CsGSs) probably also contribute to the biosynthesis in tea plants [11–13]. After synthesis, theanine is transported via the vascular system to the young shoots [2]. Six amino acid permeases (AAPs) were identified to be theanine transporters in tea plants, and CsAAP1 likely mediates theanine root-to-shoot transport [14]. Theanine degradation mainly occurs in tender leaves and was suggested to be catalyzed by CsPDX2.1 in tea plants [15].

In early spring, the theanine content in new shoots gradually increases from early March and reaches its highest level in early April. However, in late spring theanine content greatly decreases [14, 16, 17]. Along with the decrease in theanine content in new shoots in late spring, the quality of green tea processed from these new shoots also generally reduces [18] However, how the decrease in theanine content is regulated in new shoots in late-spring tea plants is not well understood.

The rapid growth of new shoots of tea plants in late spring requires a large amount of nitrogen. As one of the important storage forms of nitrogen in tea plants, theanine is hydrolyzed into glutamate and ethylamine to provide nitrogen for the growth of new shoots [19]. Therefore, theanine hydrolysis is probably regulated by shoot development and contributes to the decrease in theanine content in late-spring tea plants. However, the underlying molecular mechanism of the regulation of theanine hydrolysis in the new shoots of late-spring tea plants is largely unknown.

Although theanine is unique to tea plants, it can also be hydrolyzed by other species, including yeast, *Arabidopsis*, and tomato [14, 20]. In this study, to study the regulation of theanine hydrolysis and accumulation, we performed genetic screening of a yeast mutant library for mutants hypersensitive to 40 mM theanine. In this screening we identified 25 theanine-hypersensitive mutants, including *glutamate dehydrogenase 2* (*gdh2*). This screening implied that GDH2 participates in the regulation of theanine accumulation in tea plants. Indeed, one of the GDH2 homologs in the tea plant, CsGDH2.1, rescued the theanine-hypersensitive phenotype of *gdh2*. The subcellular localization, expression pattern, and *in vitro* enzymatic activity supported a role of CsGDH2.1 in glutamate and theanine accumulation in late-spring tea. Furthermore, the accumulation of glutamate and theanine was increased when *CsGDH2.1* expression was knocked down in the new shoots of the tea plant. Therefore, these results indicate that CsGDH2.1 negatively regulates theanine accumulation in late-spring tea shoots, probably by catalyzing glutamate catabolism.

## Results

### Yeast *gdh2* mutant was hypersensitive to a high concentration of theanine

In this study, we first performed a genetic screen on the yeast deletion mutant library for theanine-hypersensitive mutants, as previously described [14]. In this screening, we identified 25 yeast mutants that were hypersensitive to 40 mM theanine. One of these mutants, *glutamate dehydrolase 2* (*gdh2*), was of great interest, given that glutamate is both the substrate of theanine synthesis and a product of theanine degradation.

**Figure 1:**
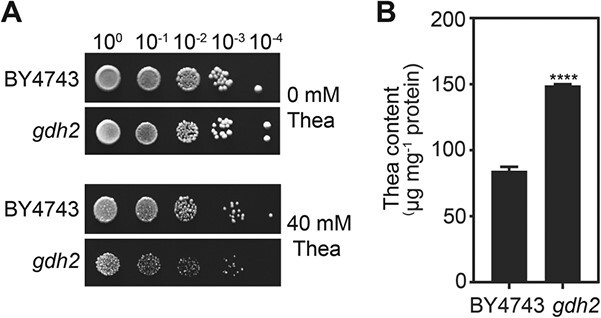
Theanine sensitivity and theanine accumulation in yeast wild type (BY4743) and *gdh2* mutant. (A) Dot assay of yeast mutant *gdh2* with 40 mM theanine. The yeast strains were cultured on YNB solid medium with or without 40 mM theanine for 3 days. *gdh2*, *glutamate dehydrogenase 2*. (B) Theanine content in yeast strains treated with 20 mM theanine for 24 hours. Data represent mean ± standard deviation (*n* = 3). Asterisks above the error bar indicate significant differences by Student’s *t*-test (^****^P ≤ .0001).

To verify the hypersensitivity of *gdh2* to theanine, a dot assay was performed. Yeast wild type BY4743 and the *gdh2* mutant were cultured on YNB medium with or without 40 mM theanine for 3 days. Results showed that the growth of wild type BY4743 was suppressed on medium containing 40 mM theanine compared with 0 mM theanine. However, the growth of *gdh2* was more severely inhibited on medium containing 40 mM theanine, and exhibited a theanine-hypersensitive phenotype (Fig. 1A).

We hypothesized that the theanine hypersensitivity of *gdh2* resulted from hyperaccumulation of theanine in this mutant. To confirm this hypothesis, yeast strains were cultured in YNB medium with 20 mM theanine for 24 hours to test theanine accumulation. The results showed that the theanine content was significantly higher in *gdh2* than in BY4743, indicating that *gdh2* accumulated more theanine than the wild type BY4743 (Fig. 1B). These results suggested that GDH2 is involved in theanine metabolism in yeast. This led us to hypothesize that the homolog of GDH2 in tea plants plays an important role in theanine accumulation.

### Identification and phylogenetic analysis of GDH2 homologs in tea plants

Generally, the similarity of yeast proteins to their homologs in plants was low. Given that GDH2 has been cloned in the model plant *Arabidopsis*, we used the protein sequence of AtGDH2 to search the GDH2 homologs in tea plant using the online BLAST program in the Tea Plant Information Archive (TPIA; tpia.teaplant.org). This search found four proteins (CSS0034454, CSS0007238, CSS0046767, and CSS0002543) having highly conserved sequences with AtGDH2. The AtGDH2 homologs in poplar (*Populus trichocarpa*), grape (*Vitis vinifera*), and rice (*Oryza sativa*) were also found using the online BLAST program (https://blast.ncbi.nlm.nih.gov/Blast.cgi).

**Figure 2:**
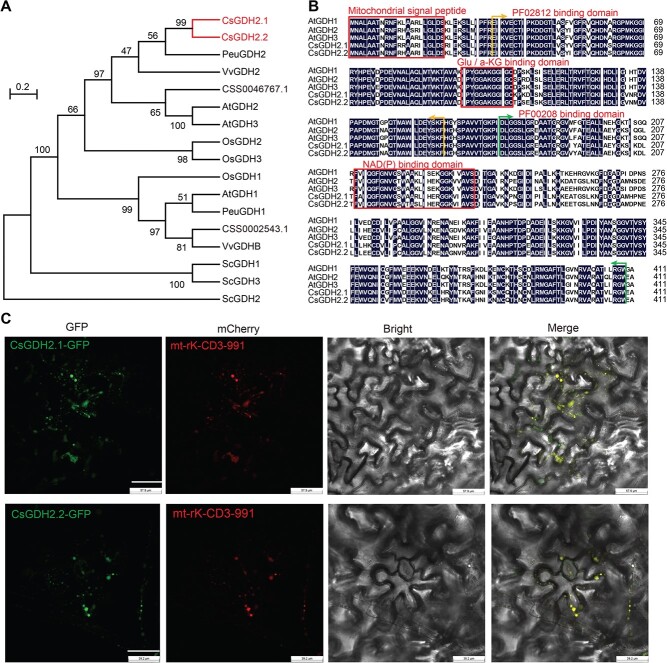
Phylogenetic analysis of GDH2 homologs and the subcellular localization of CsGDHs. (A) Phylogenetic relationship of GDHs in tea (*Camellia sinensis*), *Populus euphratica* (Peu), *Arabidopsis thaliana* (At), *Vitis vinifera* (Vv), *Oryza sativa* (Os), and *Saccharomyces cerevisiae* (Sc). The red font indicates the gene cloned in this study. (B) Sequence alignment of CsGDH2s and AtGDHs. Completely conserved residues are marked with a dark blue background; red boxes indicate conserved domains; yellow arrows indicate PF02812-binding domains; green arrows indicate PF00208-binding domains. Glu, glutamate; a-KG, α- ketoglutarate. (C) Subcellular localization of CsGDH2.1 and CsGDH2.2 in epidermal cells of tobacco leaves. GFP, CsGDH2.1–GFP, and CsGDH2.2–GFP were transiently co-expressed with mt-rK-CD3-991. Green fluorescence indicates GFP fusion protein and red fluorescence indicates mCherry-fused mitochondrial marker protein. Merged image represented overlay of green and red fluorescence.

A phylogenetic tree was constructed to analyze the evolutionary relationship of these CsGDHs in tea plant with these homologs in other plants and yeast (Fig. 2A). The phylogenetic tree showed that two CsGDHs (CSS0034454 and CSS0007238) are more conserved with GDH2 homologs in the woody plants poplar and grape. Therefore, CSS0034454 and CSS0007238 are the homologs of GDH2 in woody plants, and were named as CsGDH2.1 and CsGDH2.2, respectively. This phylogenetic tree also showed that CSS0046767 is more similar to AtGDH2, and CSS0002543 is the homolog of GDH1 in *Arabidopsis*, poplar, and grape.

Given that tea plants are woody plants and CsGDH2.1 and CsGDH2.2 are more conserved with their homologs in the woody plants poplar and grape, we chose CsGDH2.1 and CsGDH2.2 for further study. AtGDHs have been well studied in *Arabidopsis*, and we therefore aligned the protein sequences of CsGDH2.1 and CsGDH2.2 with AtGDHs. Conserved domains, including mitochondrial signal peptide, glutamate/α-ketoglutarate binding domain, and NAD(P) binding domain, were found in CsGDH2.1 and CsGDH2.2 (Fig. 2B), further suggesting that CsGDH2.1 and CsGDH2.2 are the functional homologs of GDH2 in the tea plant.

### Subcellular localization of CsGDH2.1 and CsGDH2.2

AtGDHs localize in the mitochondria of *Arabidopsis* cells [21]. CsGDH2.1 and CsGDH2.2 also have a mitochondrial signal peptide in the N terminus (Fig. 2B), and were speculated to localize in the mitochondria. To observe the subcellular localization, CsGDH2.1–GFP or CsGDH2.2–GFP fusion protein was co-expressed in tobacco epidermal cells with a mitochondrial marker protein, mt-rK-CD3-991 [22]. The fluorescence of CsGDH2.1–GFP and CsGDH2.2–GFP was observed to fully overlap with that of mt-rK-CD3-991 (Fig. 2C). These results indicated that CsGDH2.1 and CsGDH2.2 localize in the mitochondria.

### Expression of *CsGDH2.1* in yeast *gdh2* mutant rescued the theanine-hypersensitive phenotype

**Figure 3:**
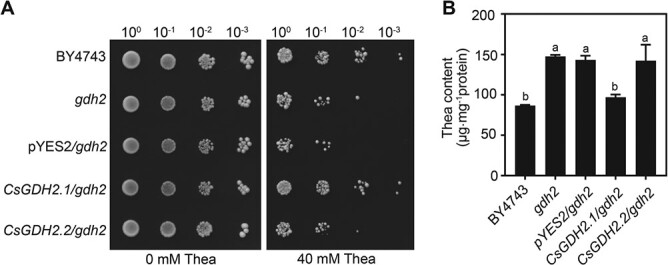
Yeast complementation assay of CsGDH2s. (A) Dot assay of yeast mutant *gdh2* with 40 mM theanine. Yeast gdh2 cell expressing empty vector (pDR196), *CsGDH2.1*, or *CsGDH2.2* grew on YNB medium containing 0 or 40 mM theanine for 3 days. (B) Theanine contents in yeast strains treated with 20 mM theanine for 24 hours. Data represent mean ± standard deviation (*n* = 3). Different letters above the error bar indicate significant differences by one-way ANOVA and Duncan’s multiple range test (*P* < .05).

To verify the role of CsGDH2.1 and CsGDH2.2 in theanine accumulation, we introduced *CsGDH2.1* and *CsGDH2.2* into the yeast *gdh2* mutant and obtained *CsGDH2.1*/*gdh2* and *CsGDH2.2*/*gdh2* strains, respectively. Yeast strains were cultured on YNB medium with or without 40 mM theanine for 3 days. As clearly shown in Fig. 3A, the growth of strain *CsGDH2.1*/*gdh2* recovered to the wild-type level on YNB medium with 40 mM theanine. In contrast, the growth of strain *CsGDH2.2*/*gdh2* was still hypersensitive to 40 mM theanine, just like that of the *gdh2* mutant and the *gdh2* transformed with empty vector pYES2 (pYES2/*gdh2*). Consistently, the expression of *CsGDH2.1* in the *gdh2* mutant also reduced theanine accumulation to the wild-type level (Fig. 3B). These results implied that CsGDH2.1 is functional in regulating theanine accumulation in tea plants. Thereafter, we focused on CsGDH2.1 to study its role in theanine accumulation.

### *CsGDH2.1* was highly expressed in young leaves of tea plants

**Figure 4:**
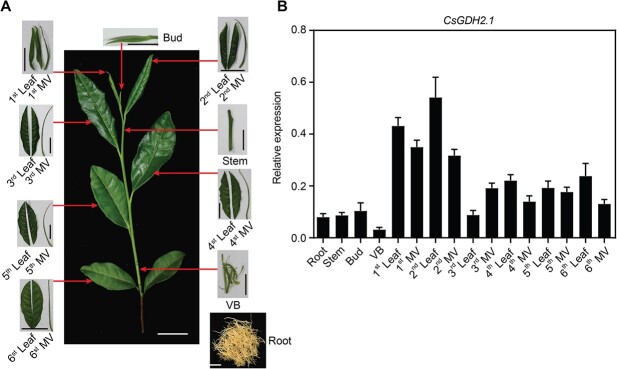
Tissue-specific expression of *CsGDH2.1* in tea plant. (A) Tissues for the analysis of *CsGDH2.1* expression. VB, vascular bundle; MV, major vein. Scale bar, 1 cm. (B) Relative expression level of *CsGDH2.1* in different tissues of tea plant. Data represent mean ± standard deviation (*n* = 3).

To explore the role of CsGDH2.1 in theanine accumulation in tea plants, we examined the expression of *CsGDH2.1* in different tissues, including root, young stem, leaf bud, vascular bundle in the stem, and developing, mature, and old leaves (including the lamina and the major vein) (Fig. 4A). The results showed that *CsGDH2.1* was highly expressed in the developing first and second leaves, and relatively less expressed in the root, stem, leaf bud, and vascular bundle in the stem (Fig. 4B). Interestingly, theanine catabolism occurs mainly in young leaves of tea plants [2], and the proposed theanine hydrolase-encoding gene *CsPDX2.1* is also highly expressed in leaves [15]. Therefore, the tissues where *CsGDH2.1* is expressed coincide with those where theanine is degraded.

### *CsGDH2.1* expression increased in late-spring tea leaves

**Figure 5:**
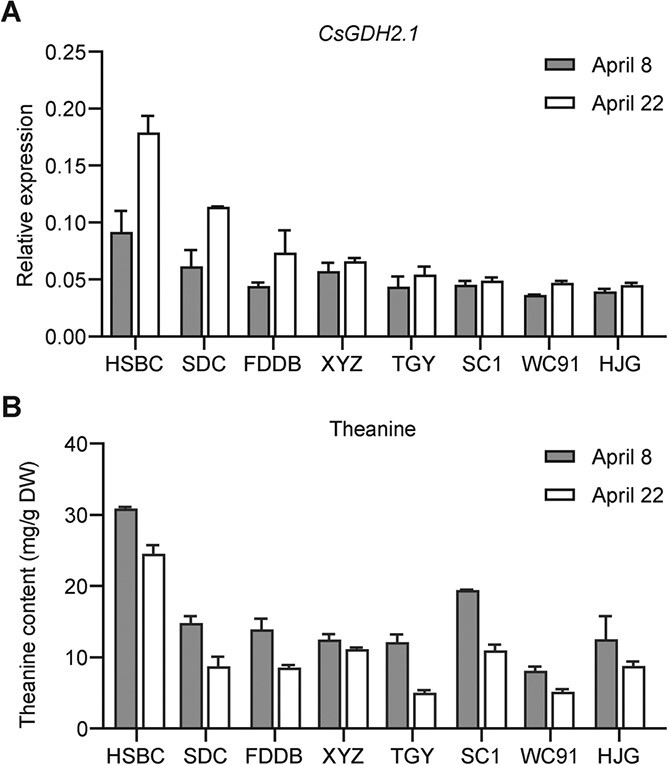
*CsGDH2.1* expression and theanine contents in the first leaf of eight tea cultivars at different time points. *CsGDH2.1* expression levels in the first leaf of ‘Huangshan Baicha’ (HSBC), ‘Shidacha’ (SDC), ‘Fuding Dabaicha’ (FDDB), ‘Xianyuzao’ (XYZ), ‘Tieguanyin’ (TGY), ‘Shancha 1’ (SC1), ‘Wancha 91’ (WC91), and ‘Huangjinya’ (HJY) at 8 April and 22 April. Data represent mean ± standard deviation of three independent biological replicates.

In our previous study, we found that theanine contents in new shoots significantly decreased in the middle of April [16]. To obtain more insight into the role of CsGDH2.1 in theanine accumulation, we next examined the expression of *CsGDH2.1* in the first leaf of eight tea plant cultivars at two time points, 8 April and 22 April. The expression of *CsGDH2.1* in the first leaf was higher at 22 April compared with that at 8 April in the all tea plant cultivars examined (Fig. 5A). Consistently, the theanine contents in all these samples was lower at 22 April (Fig. 5B). Therefore, generally, the time of induction of *CsGDH2.1* expression in late spring coincides with that of the decrease in theanine content in the leaf. The results support a negative role of *CsGDH2.1* in theanine accumulation in late-spring tea leaves.

### CsGDH2.1 had glutamate catabolic activity *in vitro*

**Figure 6:**
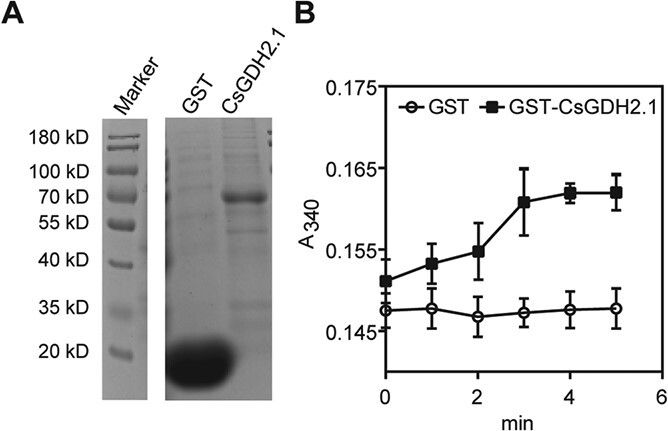
*In vitro* enzymatic activity analysis of CsGDH2.1. (A) SDS–PAGE analysis of purified CsGDH2.1. (B) Absorbance at 340 nm during the CsGDH2.1 enzymatic reaction. Data represent mean ± standard deviation (*n* = 3)

Given that glutamate is both the substrate of theanine synthesis and a product of theanine degradation, glutamate likely increases theanine accumulation in new shoots, by promoting synthesis and feedback repressing the catabolism of theanine. Based on the negative relationship between *CsGDH2.1* expression and theanine accumulation, we speculated that CsGDH2.1 functions in glutamate catabolism in the new shoots, in the period of theanine degradation. To test whether CsGDH2.1 has glutamate catabolic activity, GST-tagged CsGDH2.1 (GST-CsGDH2.1) was expressed and purified from *Escherichia coli* (Fig. 6A). Glutamate and NADPH were used as the substrates to test the *in vitro* enzymatic activity.

The activity was determined by the absorbance at 340 nm of NADH after 1, 2, 3, 4 and 5 minutes of reaction. Absorbance greatly increased when the substrates were mixed with GST-CsGDH2.1. In contrast, the absorbance remained stable when the substrates were mixed with GST control (Fig. 6B). These results indicated CsGDH2.1 had glutamate catabolic activity *in vitro*.

### Transient suppression of *CsGDH2.1* increased glutamate and theanine accumulation in the new shoot

**Figure 7:**
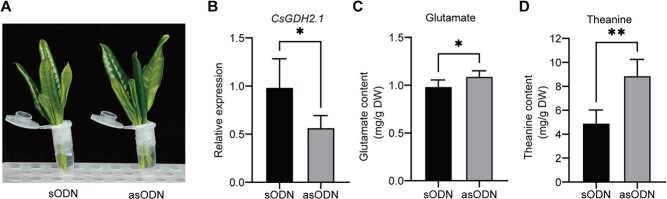
Effects of transient suppression of *CsGDH2.1* on the accumulation of glutamate and theanine. (A) New shoots of tea plants treated with sODN-*CsGDH2.1* and asODN-*CsGDH2.1*. (B) Expression of *CsGDH2.1* in new shoots of tea plants treated with sODN-*CsGDH2.1* and asODN-*CsGDH2.1*. (C) Glutamate content in new shoots of tea plants fed with asODN-*CsGDH2.1*. (C) Theanine content in new shoots of tea plants treated with sODN-*CsGDH2.1* and asODN-*CsGDH2.1*. Data represent mean ± standard deviation (*n* = 3). Asterisks above the error bar indicate significant differences by Student’s *t*-test (^*^P ≤ .05; ^**^P ≤ .01).

Currently, antisense oligonucleotide (asODN) technology is widely used to suppress gene expression in tea plants [23–25]. To test the *in vivo* role of CsGDH2.1 in glutamate and theanine accumulation, we used *CsGDH2.1*-specific asODN to treat new shoots, and used the *CsGDH2.1*-specific sense oligonucleotide (sODN) as the control (Fig. 7A). The asODN treatment significantly reduced the expression of *CsGDH2.1* in the new shoots, compared with the sODN control treatment (Fig. 7B). Under this condition, the glutamate and theanine contents significantly increased in these asODN-treated new shoots (Fig. 7C and D). These results indicated that the expression of *CsGDH2.1* negatively regulated glutamate and theanine accumulation in the new shoots. These results also supported the notion that CsGDH2.1-catalyzed glutamate catabolism reduces glutamate accumulation, and the reduction of glutamate further promotes theanine catabolism.

## Discussion

Theanine is primarily synthesized and stored in roots in winter and is transported to the new shoots, where it is degraded, in spring [2]. However, how theanine degradation is regulated in new shoots in spring, especially in the late spring, remains largely elusive. In this study, to explore the regulatory mechanism underlying the decreased theanine accumulation in tea shoots in late spring, we first performed a genetic screen on a yeast deletion mutant library of theanine-hypersensitive mutants and found that GDH2 regulated theanine accumulation in yeast. The results of subsequent experiments indicated that CsGDH2.1, the homolog of yeast GDH2 in the tea plant, also regulated theanine accumulation in the new shoots of late-spring tea plants.

Generally, GDHs catalyze reversible amination and deamination to synthesize and catabolize glutamate, respectively [26]. However, studies indicated that the main function of GDHs in plants is to catalyze the deamination of glutamate to release ammonia and α-ketoglutarate to participate in carbon and nitrogen metabolism [21, 26–29]. In tea plants, CsGDHs were suggested to be involved in NH_4_^+^ assimilation, especially under high NH_4_^+^ conditions [30]; however, evidence for the function of CsGDHs in tea plants was still circumstantial. The role of CsGDHs in glutamate catabolism in tea plants has not yet been investigated. In our previous study, we found that the expression of one *CsGDH* was negatively correlated with the theanine contents in the leaf buds of tea plants [16], suggesting a negative role of CsGDH in theanine accumulation. In this study, we further observed that the increase in *CsGDH2.1* expression in late spring coincided with the significant decrease in theanine content in the new shoots (Fig. 5), and provided evidence that CsGDH2.1 negatively regulates theanine accumulation, probably by catalyzing glutamate catabolism, in the new shoots of late-spring tea plants.

The degradation of individual amino acids was proposed to be a highly relevant process for adjusting amino acid contents [31]. GDH catalyzes the oxidative deamination of glutamate, which largely affected glutamate accumulation in plants [21, 26–29]. Interestingly, some researchers found that GDH can also affect the metabolism of other amino acids besides glutamate, such as alanine, γ-aminobutyrate, asparagine, and proline, given that glutamate provides ammonium for the biosynthesis of these amino acids [32]. Glutamate is both the product of theanine degradation and the precursor of theanine biosynthesis, so it is reasonable that CsGDH2.1-catalyzed oxidative deamination of glutamate (Fig. 6) negatively regulates theanine accumulation.

It is known that GDH-catalyzed oxidative deamination of glutamate occurs in the mitochondria [31]. As a result of glutamate catabolism, the carbohydrate skeleton of glutamate is converted to α-ketoglutarate, the intermediate of tricarboxylic acid (TCA) cycle. This process contributes to mitochondrial metabolism and the production of energy, such as ATP, NADH, and FADH2 generation [31]. Here, we also observed that CsGDH2.1 localized in the mitochondria (Fig. 2), suggesting that CsGDH2.1-catalyzed glutamate catabolism in tea plant also occurs in the mitochondria. This process is likely critical for reducing glutamate accumulation and providing nitrogen and energy for the fast growth of the new shoots of tea plants in the late spring.

The new shoots of tea plants also have theanine synthetic activity, although much lower than that in the roots [2]. Both CsTSI and glutamine synthetases (CsGSs) may contribute to theanine synthesis from glutamate and ethylamine in the new shoots [15]. So, the new shoots can degrade theanine into glutamate and ethylamine, and can also synthesize theanine using glutamate and ethylamine. Then the question becomes: how are synthesis and catabolism regulated in the new shoots? There must be a signal to turn on or off the synthesis and catabolism of theanine in the new shoots. Given the pivotal role of glutamate in the signaling of plant growth, development, and amino acid metabolism [26, 32], glutamate itself is the best candidate signal controlling theanine synthesis and catabolism in the new shoots of tea plants.

**Figure 8:**
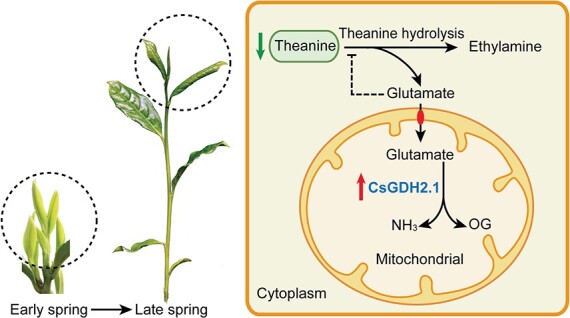
Proposed model for CsGDH2.1-regulated theanine accumulation in the new shoots of late-spring tea plants. OG, 2-oxoglutarate.

Currently, both the biosynthesis and hydrolysis of theanine are thought to occur in the cytoplasm [12, 15]. CsPDX2 is probably the theanine hydrolase that mediates theanine degradation into glutamate and ethylamine in the cytoplasm [14]. We also tested whether CsGDH2.1 can catalyze theanine hydrolysis, and the result showed that CsGDH2.1 does not have this activity *in vitro* (Supplementary Data Fig. S1). We propose that glutamate concentration in the cytoplasm ([Glu]cyt) is a signal for theanine degradation and theanine biosynthesis: when [Glu]cyt decreases, theanine degradation is promoted and theanine biosynthesis is inhibited; when [Glu]cyt increases, theanine degradation is inhibited and theanine biosynthesis is promoted (Fig. 8). While CsGDH2.1 mediated glutamate catabolism could reduce glutamate accumulation and [Glu]cyt, and therefore promotes theanine degradation (Fig. 8). Theanine is thought to be a form of nitrogen storage and transport in tea plants. Thus, CsGDH2.1-regulated theanine degradation can provide nitrogen and energy for the rapid growth of new shoots in the late spring.

To our knowledge, this is the first report focusing on the regulation of theanine accumulation in new shoots in late spring. The findings of this study are critical for optimizing cultivation technology to improve theanine content in late spring, by targeting to reduce *CsGDH2.1* expression. It is noteworthy that this study was performed in Anhui province, a representative province for the growth of tea plant variety *Camellia sinensis* var. *sinensis*. The climate of Anhui is different from that of Yunnan province of China and India, which are representative of *C. sinensis* var. *assamica* growth. To extend the knowledge gained in this study to tea plant *C. sinensis* var. *assamica*, more work is needed to be performed in Yunnan province, in future.

## Materials and methods

### Yeast strains and culture methods

Yeast theanine-hypersensitive mutant *gdh2* was screened as previously reported [14]. The composition of the culture medium was as follows: 1.7 g/l yeast base (Difco™ Yeast Nitrogen Base w/o Amino Acids and Ammonium Sulfate, BD, USA), 20 mg/l uracil, 5 g/l (NH_4_)_2_SO_4_, 20 g/l galactose, 20 g/l agar powder (for solid medium only). The medium was adjusted to pH 5.5–6.0 using KOH and autoclaved at 121°C for 20 minutes. For 40 mM theanine treatment, 1 M theanine (Lanji Technology, Shanghai, China) solution was filtered with a 0.22 μm Sterile PES Syringe Filter (Sorfa Life Science Research, Zhejiang, China) before adding to the culture medium. The pre-cultured yeast wild type BY4743 and the *gdh2* mutant were diluted 10-, 100-, and 1000-fold, with 2 μl of each dilution spotted on solid medium, and then cultured in an incubator at 30°C for 3 days.

### Measurement of theanine accumulation in yeast

Yeast strains were streaked on YNB medium and cultured at 30°C for 2 days to obtain colonies. The colonies of yeast strains were cultured in YNB liquid medium at 30°C to an OD_600_ of 0.8, and then were centrifuged at 8000 rpm for 2 minutes to collect yeast cells. The cells were re-suspended in YNB liquid medium containing 20 mM theanine to an OD_600_ of 0.5, and then cultured with a rocker at 30°C for 24 hours. The yeast cells were collected by centrifugation and washed twice with 3 ml buffer (0.6 M sorbitol, 50 mM potassium phosphate, pH 4.5), and then washed with 3 ml distilled water. The yeast precipitate was re-suspended with 1 ml distilled water and heated in a water bath at 100°C for 30 minutes to extract theanine. After centrifugation at 12 000 rpm for 30 minutes, the supernatant was passed through a 0.22 μm filter for the subsequent HPLC-based measurement of theanine content.

### Yeast complementation assay

The wild-type strain BY4743 and the *gdh2* mutant strain were used for a complementation assay. *CsGDH2.1* and *CsGDH2.2*, cloned in the pDR196 vector, were introduced into the *gdh2* mutant. The *gdh2* mutant transformed with pDR196 was used as a negative control. Yeast strains were cultured on YNB medium containing 0 or 40 mM theanine at 30°C for 3 days.

### Gene cloning and sequence analysis

The sequences of *CsGDH2.1* and *CsGDH2.2* were retrieved from the tea genome database TPIA (tpia.teaplant.org) [33], and were amplified by PCR. The primers used for gene cloning are listed in Supplementary Data Table S1. The PCR product was inserted into yeast pYES2. Multiple sequence alignment of full-length GDH proteins was completed by ClustalW and a phylogenetic tree was constructed by MEGAX software. Protein sequence alignment of GDHs was performed by DNAMAN.

### *CsGDH2* expression analysis

Samples used for the expression analysis of *CsGDH2* were collected from tea plants grown at Guohe tea plantation, Anhui, China. Tissues were frozen in liquid nitrogen and stored at −80°C after being collected. Total RNA was extracted from different tissues of tea plant cultivar ‘Shuchazao’ for the tissue-specific expression analysis of *CsGDH2.1*. The tissues include root, bud, stem, vascular bundles, the first, second, third, fourth, fifth and sixth leaves, and the major vein.

For the analysis of *CsGDH2.1* in the first leaf of various tea plant varieties, the total RNA was extracted from the first leaf of eight tea plant cultivars. These cultivars were ‘Huangshan Baicha’ (HSBC), ‘Shidacha’ (SDC), ‘Fuding Dabaicha’ (FDDB), ‘Xianyuzao’ (XYZ), ‘Tieguanyin’ (TGY), ‘Shancha 1’ (SC1), ‘Wancha 91’ (WC91), and ‘Huangjinya’ (HJY). These tea plants grew at Guohe tea plantation, Anhui, China.

Total RNA was extracted using an RNAprep Pure Plant Plus Kit (Tiangen, Beijing, China) according to the manufacturer’s instructions. The cDNAs were synthesized using the PrimeScript RT Reagent Kit (Takara, Dalian, China). qRT–PCR was performed on a QuantStudio 6 Flex Real-Time System with SYBR Green I dye (Vazyme, China). *CsGAPDH* was the internal reference gene. All primers used are listed in Supplementary Data Table S1. The data presented are mean ± standard deviation for three independent biological replicates. The 2^-△Ct^ method was used for relative gene expression calculation.

### Subcellular localization analysis of CsGDH2

The plasmid used for CsGDH2 subcellular localization was constructed using Gateway cloning technology according to the manufacturer’s instructions (Invitrogen, USA). To obtain the entry vectors, full-length *CsGDH2.1* and *CsGDH2.2* were amplified using attB-flanked primers and PrimeSTAR Max DNA Polymerase (Takara, Dalian, China) before recombination into the pDONR vector by the BP reaction. pK7WGF:GFP was used as a destination vector in the LR reaction to generate pK7WGF:CsGDH2.1–GFP and pK7WGF:CsGDH2.2–GFP, which were transformed into *Agrobacterium tumefaciens* strain EHA105 and then transiently expressed in tobacco epidermal cells together with mt-rK-CD3-991, a mitochondria marker [22].

### Theanine content determination

Theanine was extracted with distilled water as previously reported [14], with the following modifications: 100 mg of freeze-dried sample powder was dissolved in 3 ml distilled water and heated in a water bath at 100°C for 30 minutes. After centrifugation at 13 000 rpm for 20 minutes, the supernatant was passed through a 0.22 μm filter for subsequent HPLC-based analysis of theanine content. The detection conditions of HPLC analysis were as previously described [14].

### Recombinant expression, purification, and *in vitro* enzymatic reaction of CsGDH2.1

The cDNA of *CsGDH2.1* was amplified using primers with the EcoRI and NcotI restriction enzyme sites. The product was cloned into expression vector pGEX-4 T-1 using the corresponding restriction enzymes. After verification, the expression plasmids and empty vector were transformed into *E. coli* BL21 (DE3). GST and CsGDH2.1-GST were purified using GST-binding resin. α-Ketoglutarate and NADPH were used for detecting GDH reduction activity. The activity was determined by absorbance at 340 nm of NADH.

### Transiently suppression of *CsGDH4* expression in new shoots of tea plants using antisense oligonucleotides

The asODN assay was performed as previously described [22]. Candidate ODN sequences were obtained from Soligo software (http://sfold.wadsworth.org/cgi-bin/soligo.pl) with *CsGDH2.1* as input sequence. New shoots of tea plants were treated with 500 μl of 20 μM asODN solution, and sense oligonucleotides (sODN) were used as control. After being incubated for 24 hours, the first leaves were collected and stored at −80°C for further analysis. The sequence of sODN is 5′-TGGTGGAGCTAAGGGTGGAA-3′, and the asODN sequence is 5′-TTCCACCCTTAGCTCCACCA-3′.

### Statistical analysis

All statistical analysis was performed using SPSS (v19.0) software. Data represent the mean ± standard deviation of three independent biological replicates. Data were analyzed by Student’s *t*-test among three independent biological replicates, with *P* < .05 indicating a significant difference.

## Acknowledgements

This work was supported by grants from the National Natural Science Foundation of China (32072624), the National Key R&D Program of China (2021YFD1601101), and Anhui Provincial Department of Human Resources and Social Security (2021LXC017).

## Author contributions

Z.Z. and X.W. conceived the research and finalized the manuscript. T.C., J.M., H.L., S.L., C.D., Y.X., X.Y., S.Z., and T.Y. carried out the experiments and analyzed the data; T.C. and Z.Z. wrote the manuscript. Z.Z. and X.W. finalized the manuscript. All authors read and approved the manuscript.

## Data availability

All relevant data in this study are provided in the article and its supplementary file.

## Conflict of interest

The authors declare that they have no conflict of interest.

## Supplementary data

Supplementary data is available at *Horticulture Research* online.

## Supplementary Material

Web_Material_uhac245
